# Fast computation of distance estimators

**DOI:** 10.1186/1471-2105-8-89

**Published:** 2007-03-13

**Authors:** Isaac Elias, Jens Lagergren

**Affiliations:** 1 Dept. of Numerical Analysis and Computer Science, Royal Institute of Technology, Stockholm, SE-106 91, Sweden

## Abstract

**Background:**

Some distance methods are among the most commonly used methods for reconstructing phylogenetic trees from sequence data. The input to a distance method is a distance matrix, containing estimated pairwise distances between all pairs of taxa. Distance methods themselves are often fast, e.g., the famous and popular Neighbor Joining (NJ) algorithm reconstructs a phylogeny of *n *taxa in time *O*(*n*^3^). Unfortunately, the fastest practical algorithms known for Computing the distance matrix, from *n *sequences of length *l*, takes time proportional to *l*·*n*^2^. Since the sequence length typically is much larger than the number of taxa, the distance estimation is the bottleneck in phylogeny reconstruction. This bottleneck is especially apparent in reconstruction of large phylogenies or in applications where many trees have to be reconstructed, e.g., bootstrapping and genome wide applications.

**Results:**

We give an advanced algorithm for Computing the number of mutational events between DNA sequences which is significantly faster than both Phylip and Paup. Moreover, we give a new method for estimating pairwise distances between sequences which contain ambiguity Symbols. This new method is shown to be more accurate as well as faster than earlier methods.

**Conclusion:**

Our novel algorithm for Computing distance estimators provides a valuable tool in phylogeny reconstruction. Since the running time of our distance estimation algorithm is comparable to that of most distance methods, the previous bottleneck is removed. All distance methods, such as NJ, require a distance matrix as input and, hence, our novel algorithm significantly improves the overall running time of all distance methods. In particular, we show for real world biological applications how the running time of phylogeny reconstruction using NJ is improved from a matter of hours to a matter of seconds.

## Background

Today's, and tomorrow's even greater, availability of sequence data provides unprecedented opportunities to understand evolutionary relations; however, the increasing wealth of data also poses challenges to computational methods for data analysis and, thereby also, to algorithm design. The number of sequences involved in an analysis may be very large, since a large number of species or a large gene family may be considered – some species harbor hundreds of members of some gene families. The ultimate goal in species tree construction is to obtain the tree of life, to the extent it exists. As the size of the phylogenies being reconstructed grows, the demand for long sequences increases, which often is satisfied by concatenation of genes. In other applications many trees have to be reconstructed. For instance, comparative genomics studies often involves constructing gene trees for all gene families in a number of genomes, i.e., often thousands of families. Also bootstrapping, which commonly is used to obtain significance values for a single family, requires many reconstructions.

There are two major approaches to phylogenetic tree reconstruction; distance methods and character based methods (such as likelihood and parsimony). Although this paper is concerned with tree reconstruction through distance methods, character based methods are many times the preferred approach. However, since most distance methods are several orders of magnitude faster than character based methods, distance methods are preferred when it comes to extensive evolutionary analysis.

In general, distance methods first estimate pairwise evolutionary distances of the species, represent them using a distance matrix, and, then, compute the tree, or attempt to compute the tree, that realizes the given distance matrix as well as possible. The Neighbor Joining algorithm (NJ) [1] is a distance method, running in time Θ(*n*^3^), where *n *is the number of sequences, which due to its accuracy and speed has been embraced by the phylogenetic Community. However, prior to running the distance method the distance matrix has to be computed from the sequences. Although the distance matrix in theory can be computed in time *O*(*l*·*n*^1.376^), where *l *is the sequence length, see [2], the only practical algorithms require time Ω(*l*·*n*^2^). Therefore, the overall running time of tree reconstruction with NJ is Ω(*l*·*n*^2 ^+ *n*^3^). Since the sequence length often is larger than the number of taxa, Computing the distance matrix is the bottleneck. In this paper, we remove this bottleneck by presenting an algorithm which in practice has running time comparable to N.J.

There are various Markov models of sequence evolution describing how sites evolve, typically, independently and identically from the root down toward the leaves. For example, Kimura's two-parameter model (K2P) [3] distinguishes between two types of events: *transitions *which are changes within the purine (A and G) and pyrimidine (C and T) groups and *transversions *which are changes between the groups. The aim in distance estimation is to find the most likely (ML) estimate of the actual number of mutational events given the number of observed events. For the Kimura two-parameter model, this is done by counting the number of transitions and transversions between each pair of sequences and thereafter optimizing the likelihood function, using a closed formula, to attain the corrected distances. The straightforward way of counting the number of observed events between two sequences of length *l *takes Ω(*l*) time. Therefore, the straightforward algorithm of Computing all pairwise distances between *n *sequences has running time Θ(*l*·*n*^2^).

In this paper, a novel divide and conquer algorithm for Computing the number of observed events is presented. The algorithm computes the same function and has the same asymptotic running time as the straightforward algorithm. In practice, however, it is significantly faster than Phylip [4] and Paup [5]. We show on both simulated and real biological data how our algorithm speeds up the reconstruction using NJ from a matter of many minutes and even hours to a matter of seconds. It is important to note that, since the computation of the distance matrix is a prerequisite for all distance methods, our algorithm provides an increase in speed for all distance methods, i.e., not only NJ.

In addition, we present two new methods for handling, so called, ambiguity Symbols, i.e., bases of uncertain identity. Such symbols occur naturally in the form of single nucleotide polymorphisms (SNPs) and also as a result of failure to resolve bases during sequencing. Ambiguity symbols complicates matters in distance estimation and it is not obvious how to include them in the likelihood computations. We show on simulated data that our new methods for ambiguity symbols are significantly more accurate than earlier methods. This is, to the best of our knowledge, the first work which evaluates the accuracy of different ambiguity approaches.

## Results and discussion

### A novel algorithm for computing the number of mutational events

In distance estimation, a specific model of sequence evolution is used to derive an estimate of the true mutational distance between two sequences from the number of observed mutational events. Once the observed mutational events have been counted, the ML estimate of the true mutational distance is computed in constant time, either by using a closed correction formula or by using an iterative method for optimizing the likelihood function. Our novel algorithm takes two DNA sequences as input and counts the number of purine-transitions, pyrimidine-transitions, and transversions. These events are sufficient for Computing the estimates with respect to the four most common models of sequence evolution: the *Jukes-Cantor model *[6], *Kimura's two-parameter model *[3], the *Felsenstein-84 model *[7], and the *Tamura-Nei model *[8].

Our algorithm is based on an elaborate divide and conquer and bit-fiddling strategy, i.e., the problem is divided into subproblems which are solved and the Solutions are combined using the bitwise representation and low-level tricks. It is important to note that our algorithm computes the number of observed events exactly. In other words, it solves the same computational problem, i.e., it computes exactly the same value as previous algorithms for Computing the distance estimators; however, it does so significantly faster. In the remainder of this section, we show using both simulated and real biological data, that the speed of our algorithm, fastdist, outshines that of the distance estimation implemented in both Phylip [4]and Paup [5]. Although this implies that fastdist is a useful tool it does not exclude the possibility that other implementations of the naive approach can be faster. We therefore implemented our own optimized version of the naive approach and compared also its running time with fastdist.

#### Running time on simulated data – the hidden factor

The computational complexity of our distance estimation algorithm is *O*(*n*^2^·*l*), where *n *is the number of species and *l *the sequence length. This is the same complexity as that of the algorithm in the Phylip package and that of our implementation of the naive approach. However, the differences between the hidden constant in the big-O expressions of the methods are very significant. To estimate the ratio between the hidden constants, we ran the programs (The programs were run on a PowerMac dual Dual-core Xeon (Woodcrest), 2.66 GHz, 4 Mb cache per chip, 2 Gb dual channel RAM, 1066 MHz front side bus.) on datasets of 100 taxa and varied the sequences length. The test results, which are displayed in Table 1, show that fastdist is a factor 8.5–11.5 times faster than our implementation of the naive approach and a factor 180–300 times faster than Phylip, depending on whether reading the sequences is included in the comparison. With respect to Paup our algorithm is about 90 times faster on sequences of length 10,000, about 900 times faster on sequences of length 100,000, and more than 8000 times faster on sequences of length l million. It turns out that Paup adopts a preprocessing that has runtime complexity *O*(*n*^2^·*l*^2^) (David Swofford intends to make this preprocessing more efficient). Also notice that our own implementation of the naive algorithm is significantly faster than both Phylip and Paup.

**Table 1:** Runtime comparison on simulated data.

**Seq. Length**	**Phylip**	**Paup**	**Naive**	**Fastdist**
10,000	5.47 s	2.69 s	0.24 s	0.03 s
100,000	60.2 s	4 m25 s	2.32 s	0.27 s
1,000,000	-	371 m50 s	23.2 s	2.71 s

Comparing the running time o Phylip s dnadist, Paup, our optimized Implementation of the naive approach, and the new algorithm fastdist. The test's are run on simulated data with 100 species. It should be noted that Phylip could not be run on sequences of length 1,000,000. Except for Paup which uses the analytic formula, the programs computed the K2P distance with a globally fixed transition transversion ratio of 2. Reconstructing a tree of 100 taxa using NJ takes no more than 0.01 seconds. Moreover, simply reading a file containing 100 sequences of length one million with the wc unix utility takes 0.75 seconds.

Running Neighbor Joining on a distance matrix of 100 taxa takes no more than 0.01 seconds. Therefore, even when a fairly moderate sequence length of 10,000 nucleotides is considered, overall, it takes 0.04 seconds to reconstruct the tree using fastdist+NJ, 5.48 seconds using Phylip+NJ, and 2.70 seconds with Paup+NJ.

#### Running time on real biological data

Above we reported experiments on large simulated datasets. At present there are, however, no real biological datasets available of such sizes. We have selected two large datasets from the literature; one containing 29 RNA sequences of length 9168 from the Hepatitis C Virus [9] and one containing 146 copies of a nuclear gene in birds with a sequence length of 2974 [10]. We decided to perform 1000 bootstrap evaluations for both datasets. As can be seen in Table 2, our new algorithm is significantly faster than Phylip on both datasets. The reader should notice that fastdist+NJ performs the task in a matter of seconds while Phylip+NJ takes many minutes or even hours. Also notice that although our own implementation of the naive algorithm is slower than fastdist it is still much faster than Phylip.

**Table 2:** Runtime comparison on biological data.

**Dataset**	**Phylip**	**Naive**	**Fastdist**	**NJ**
Birds [10]	119 m40 s	257.4 s	35.12 s	20.4 s
Hepatitis C [9]	23 m	25.9 s	6.37 s	0.35 s

Comparing the running time of Phylip's dnadist, our optimized implementation of the naive approach, and the new algorithm fastdist (without the resolution technique) on real world biological datasets when 1000 bootstrap evaluations were performed. The NJ column describes the time it takes to reconstruct the 1000 trees using the implementation of [2]. The dataset Birds [10] contains 146 copies of a nuclear gene in birds with a sequence length of 2974. The dataset Hepatitis C [9] contains 29 RNA sequences of length 9168 from the Hepatitis C Virus. Using the wc unix utility for reading the file containing 1000 bootstrapped datasets of [10] takes 4.35 seconds and reading the datasets of [9] takes 2.66 seconds.

Unfortunately, it is not possible to run Paup on a file containing multiple datasets. However, it seems reasonable to assume, considering its performance on simulated datasets, that Paup should have approximately half the running time of Phylip.

### A novel method of handling ambiguity Symbols

*Ambiguity Symbols*, i.e., bases of uncertain identity, complicates matters in sequence comparison. Such Symbols occur naturally in the form of single nucleotide polymorphisms (SNPs) and also as a result of failure to resolve bases during sequencing. Even though ambiguity Symbols are rare they are usually not omitted from the analysis but instead treated in one of two ways, either as Swofford [5] or as Felsenstein [11]. We suggest two novel methods for handling ambiguity Symbols: one general method for estimating pairwise distances and one method for adjusting the probability distribution of the resolutions in each ambiguity. The default in our program fastdist is to combine these two methods.

We evaluated the accuracy of the four methods: Swofford [5], Felsenstein [11], our general method, and the default in fastdist. As detailed in the Methods section, we first generated sequence data without ambiguities and thereafter we inserted ambiguity Symbols at random. The accuracy was then measured by Computing matrix norms (*L*_1_, *L*_2_, and *L*_∞_) between the ML estimate of the data without ambiguities and the matrices computed by the four different methods on the data with ambiguities.

**Figure 1:**
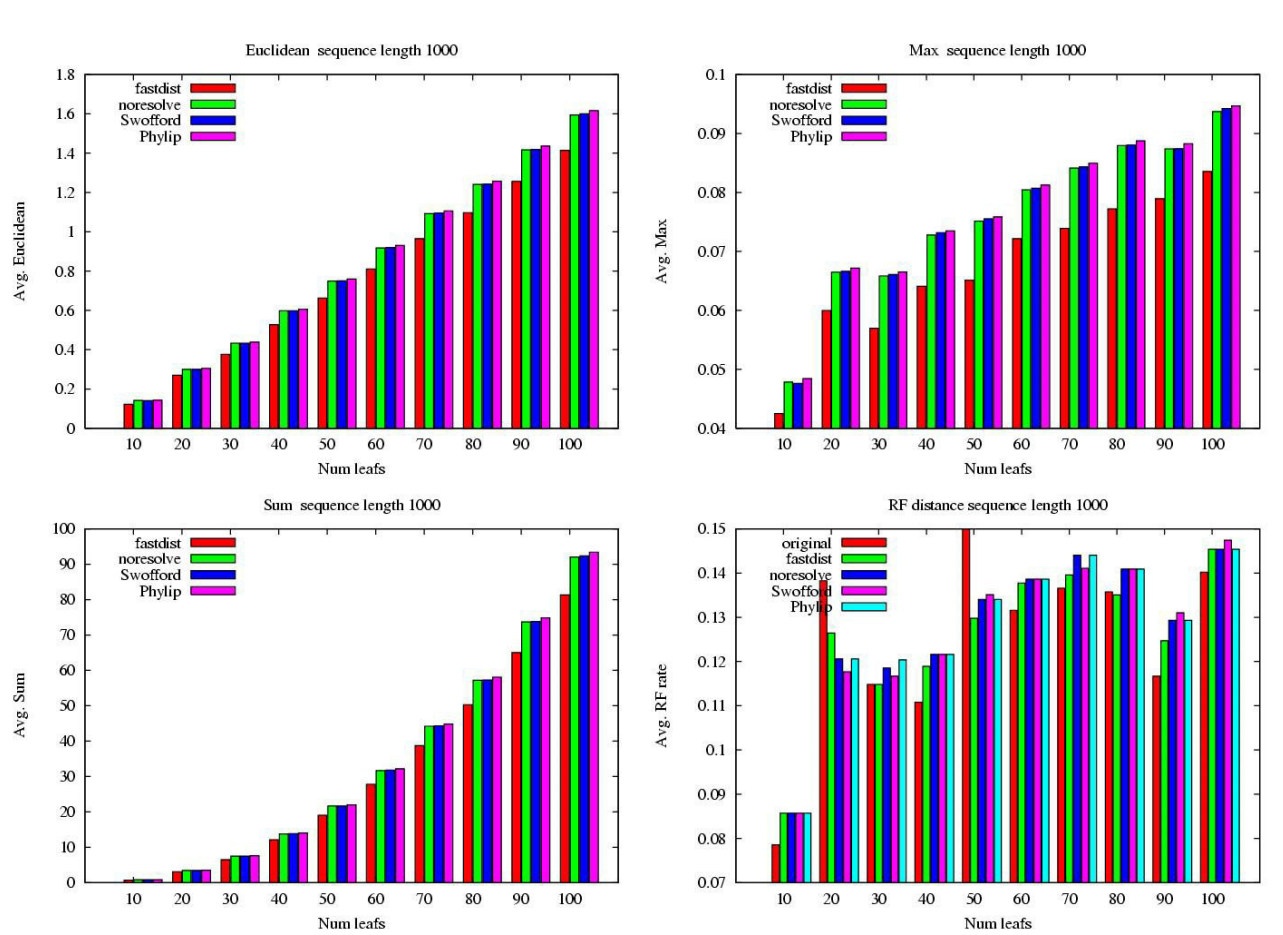
**Accuracy of ambiguity approaches**. Comparison of four different methods for handling ambiguity Symbols on sequences of length 1000 with 2% ambiguities inserted uniformly at random. Each method was used to compute a distance matrix for the data containing ambiguities. Thereafter, three different matrix norms were computed from the distance matrices computed by the methods to the correct distance matrix, i.e., the matrix computed from the data before ambiguities were inserted. The fourth graph shows the Robinson-Foulds distance between the model tree and the Neighbor Joining trees computed from the different distance matrices. To get statistically sound results the average was taken over 20 runs. fastdist is the combined method described in Figure 3. noresolve is the general technique without nearest neighbor resolution. Swofford is the method suggested by Swofford. Phylip is the Output from the Phylip package which uses Felsenstein's approach. original represents the accuracy of NJ on the data before the ambiguities were inserted (notice how NJ for some cases is more accurate on data with ambiguities).

As can be seen in Figure 1, the fastdist default, denoted fastdist in the figure, is significantly more accurate than the other three methods. Our general method, denoted noresolve, and Swofford's method have almost identical accuracy. Felsenstein's method, denoted Phylip, is slightly less accurate than Swofford's and our general method. Although Swofford's approach and our general method have similar accuracy our method requires less information and time to be computed. In particular, our method for handling ambiguities can be used together with our divide and conquer algorithm for fast computation of observed mutational events.

## Conclusion

Reconstruction of phylogenies with distance methods, such as NJ, is done by first Computing a distance matrix and thereafter applying the distance method. While NJ and other distance methods are fast, the computation of the distance matrix is slow. In this paper, we provide a very fast algorithm for Computing the distance matrix. This algorithm provides a valuable tool in tree reconstruction by improving the overall running time of distance methods by a huge factor; compared to both Phylip and Paup. We also present two novel methods for handling ambiguity symbols which, when combined together, provide significantly more accurate estimation than earlier methods.

The improvement our new algorithm gives is especially apparent in genome wide applications or when performing significance testing through bootstrapping. Using our algorithm it is possible to perform extensive bootstrapping in a matter of seconds, even for very large datasets. This facilitates extensive bootstrapping in other applications, e.g., for Computing initial guesses for other types of reconstruction algorithms, such as likelihood heuristics.

## Methods

### Supported models of sequence evolution

Our novel divide and conquer algorithm for distance estimation takes two DNA sequences as input and counts the number of purine-transitions, pyrimidine-transitions, and transversions. The number of such events are sufficient for estimating distances with respect to the four most common models of sequence evolution: the *Jukes-Cantor model *[6], *Kimura's two-parameter model *[3], the *Felsenstein-84 model *[7], and the *Tamura-Nei model *[8]. In this paper, the algorithms are presented with respect to Kimura's two-parameter model. However, all methods are generalizable also to the Tamura-Nei model, which is the most general model supported by the divide and conquer algorithm. We briefly introduce Kimura's two-parameter model and refer the reader to Felsenstein's phylogeny book [11] for an excellent exposition on distance estimators, as well as phylogeny in general.

**Figure 2:**
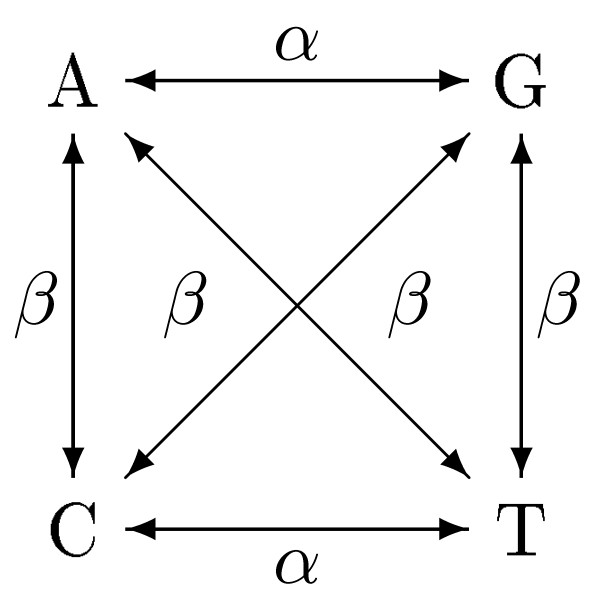
**Kimura's two-parameter model**. Kimura's two-parameter model, with *α *as the transition probability and *β *as the transversion probability. In the Jukes-Cantor model *α *= *β*.

Kimura's two-parameter model [3], Figure 2, is a generalization of the Jukes-Cantor model. This model distinguishes between two types of state transitions; *transitions *which are changes within the purine (A and T) and pyrimidine (C and G) groups and *transversions *which are changes between the groups. The following is a closed formula for the ML estimate of the evolutionary distance between two sequences *s*_1 _and *s*_2 _with respect to the Kimura two-parameter model

K2P(s1,s2)=12⋅ln⁡(11−2P−Q)+14⋅ln⁡(11−2Q),
 MathType@MTEF@5@5@+=feaafiart1ev1aaatCvAUfKttLearuWrP9MDH5MBPbIqV92AaeXatLxBI9gBaebbnrfifHhDYfgasaacH8akY=wiFfYdH8Gipec8Eeeu0xXdbba9frFj0=OqFfea0dXdd9vqai=hGuQ8kuc9pgc9s8qqaq=dirpe0xb9q8qiLsFr0=vr0=vr0dc8meaabaqaciaacaGaaeqabaqabeGadaaakeaacqWGlbWscqaIYaGmcqWGqbaucqGGOaakcqWGZbWCdaWgaaWcbaGaeGymaedabeaakiabcYcaSiabdohaZnaaBaaaleaacqaIYaGmaeqaaOGaeiykaKIaeyypa0ZaaSaaaeaacqaIXaqmaeaacqaIYaGmaaGaeyyXICTagiiBaWMaeiOBa42aaeWaaeaadaWcaaqaaiabigdaXaqaaiabigdaXiabgkHiTiabikdaYiabdcfaqjabgkHiTiabdgfarbaaaiaawIcacaGLPaaacqGHRaWkdaWcaaqaaiabigdaXaqaaiabisda0aaacqGHflY1cyGGSbaBcqGGUbGBdaqadaqaamaalaaabaGaeGymaedabaGaeGymaeJaeyOeI0IaeGOmaiJaemyuaefaaaGaayjkaiaawMcaaiabcYcaSaaa@5790@

where *P *and *Q *are the observed rates of transitions and transversions, respectively, between the two sequences. One common technique in phylogenetic analysis is to fix the transition/transversion ratio. This is often a good idea, since all sequences have evolved under the same model and, therefore, a greater overall consistency can be achieved between different pairwise comparisons. Unfortunately, there are no known closed formulas for estimating the distance when the ratio is fixed. Instead, the ML distance is computed using iterative methods. We investigated three such methods; binary search, secant-method, and Newton-Raphson, and have found that the secant-method is the more efficient.

### New methods for handling ambiguity Symbols

#### Earlier methods for handling ambiguity Symbols

To the best of our knowledge, there are two methods for handling ambiguity Symbols; that of Swofford [5] and that of Felsenstein [11]. The former can be explained by the following example. Suppose that a site has an A in one sequence and an R, representing a purine, in the other sequence. Suppose, further, that there are 90 sites in which an A is aligned with an A and that there are 10 sites in which an A is aligned with a G. Swofford's method is to have the ambiguous site contribute with 10/100 = 0.1 to the number of observed purine-transitions, i.e. *A *↔ *G *Thereafter, the estimated distance is given by optimizing the likelihood function. Felsenstein's method deals with the ambiguities directly by incorporating them in the likelihood function. The likelihood in a site is given by the joint probability of all possible resolutions of an ambiguity. The ML distance is then given in the regular way by optimizing the likelihood function.

#### A new general method for handling ambiguity Symbols

Our novel general method for handling ambiguity Symbols proceeds in two steps. The first step is to compute the ML estimate of the model parameters based on the unambiguous sites only. In Kimura's two-parameter model there are two parameters to compute; the probability of transition, *tα*, and the probability of transversion, *tβ*, where *α *and *β *are from Figure 2 and *t *is the time. To do this, we first count the number of occurring transition and transversions. The model parameters can thereafter be estimated by optimizing the likelihood function. Once the ML-estimates of *tα *and *tβ *have been computed it is possible to compute the probability of observing the two types of mutational events: the probability of observing a transversion,

potv=12(1−e−4tβ),
 MathType@MTEF@5@5@+=feaafiart1ev1aaatCvAUfKttLearuWrP9MDH5MBPbIqV92AaeXatLxBI9gBaebbnrfifHhDYfgasaacH8akY=wiFfYdH8Gipec8Eeeu0xXdbba9frFj0=OqFfea0dXdd9vqai=hGuQ8kuc9pgc9s8qqaq=dirpe0xb9q8qiLsFr0=vr0=vr0dc8meaabaqaciaacaGaaeqabaqabeGadaaakeaacqWGWbaCdaWgaaWcbaGaem4Ba8MaemiDaqNaemODayhabeaakiabg2da9maalaaabaGaeGymaedabaGaeGOmaidaaiabcIcaOiabigdaXiabgkHiTiabdwgaLnaaCaaaleqabaGaeyOeI0IaeGinaqJaemiDaqhcciGae8NSdigaaOGaeiykaKIaeiilaWcaaa@4085@

and the probability of observing a transition,

pots=14(1−2e−2t(α+β)+e−4tβ).
 MathType@MTEF@5@5@+=feaafiart1ev1aaatCvAUfKttLearuWrP9MDH5MBPbIqV92AaeXatLxBI9gBaebbnrfifHhDYfgasaacH8akY=wiFfYdH8Gipec8Eeeu0xXdbba9frFj0=OqFfea0dXdd9vqai=hGuQ8kuc9pgc9s8qqaq=dirpe0xb9q8qiLsFr0=vr0=vr0dc8meaabaqaciaacaGaaeqabaqabeGadaaakeaacqWGWbaCdaWgaaWcbaGaem4Ba8MaemiDaqNaem4Camhabeaakiabg2da9maalaaabaGaeGymaedabaGaeGinaqdaaiabcIcaOiabigdaXiabgkHiTiabikdaYiabdwgaLnaaCaaaleqabaGaeyOeI0IaeGOmaiJaemiDaqNaeiikaGccciGae8xSdeMaey4kaSIae8NSdiMaeiykaKcaaOGaey4kaSIaemyzau2aaWbaaSqabeaacqGHsislcqaI0aancqWG0baDcqWFYoGyaaGccqGGPaqkcqGGUaGlaaa@4CFF@

It follows that the probability of not observing a change is

*p*_*oid *_= 1 - *p*_*otv *_- *p*_*ots*_.

In the second step, we compute the number of observed transitions and transversions by taking also the ambiguous sites into account. Each ambiguity symbol is represented by a prior distribution of four probabilities, one for each nucleotide. In Kimura's two-parameter model, where all nucleotides are equally likely, it is natural to use a prior which is uniform on the possible resolutions of the ambiguity symbol (Notice how this representation allows for non-uniform distributions of the probabilities such as in the Tamura-Nei model. This is also utilized by our resolution technique below.). For example, the ambiguity symbol R is represented by the prior distribution {*p*_*A *_= 0.5, *p*_*C *_= 0, *p*_*G *_= 0.5, *p*_*T *_= 0}.

Now assume that we would like to compute the contribution of one ambiguous site to the observed number of events. We denote by d1x
 MathType@MTEF@5@5@+=feaafiart1ev1aaatCvAUfKttLearuWrP9MDH5MBPbIqV92AaeXatLxBI9gBaebbnrfifHhDYfgasaacH8akY=wiFfYdH8Gipec8Eeeu0xXdbba9frFj0=OqFfea0dXdd9vqai=hGuQ8kuc9pgc9s8qqaq=dirpe0xb9q8qiLsFr0=vr0=vr0dc8meaabaqaciaacaGaaeqabaqabeGadaaakeaacqWGKbazdaqhaaWcbaGaeGymaedabaGaemiEaGhaaaaa@3093@ and d2x
 MathType@MTEF@5@5@+=feaafiart1ev1aaatCvAUfKttLearuWrP9MDH5MBPbIqV92AaeXatLxBI9gBaebbnrfifHhDYfgasaacH8akY=wiFfYdH8Gipec8Eeeu0xXdbba9frFj0=OqFfea0dXdd9vqai=hGuQ8kuc9pgc9s8qqaq=dirpe0xb9q8qiLsFr0=vr0=vr0dc8meaabaqaciaacaGaaeqabaqabeGadaaakeaacqWGKbazdaqhaaWcbaGaeGOmaidabaGaemiEaGhaaaaa@3095@ the prior distributions of the two ambiguity symbols in the ambiguous site, i.e., d1A
 MathType@MTEF@5@5@+=feaafiart1ev1aaatCvAUfKttLearuWrP9MDH5MBPbIqV92AaeXatLxBI9gBaebbnrfifHhDYfgasaacH8akY=wiFfYdH8Gipec8Eeeu0xXdbba9frFj0=OqFfea0dXdd9vqai=hGuQ8kuc9pgc9s8qqaq=dirpe0xb9q8qiLsFr0=vr0=vr0dc8meaabaqaciaacaGaaeqabaqabeGadaaakeaacqWGKbazdaqhaaWcbaGaeGymaedabaGaemyqaeeaaaaa@3025@ represents the probability of observing an A in the ambiguous site of first sequence. The posterior probability of each mutational event can then be computed as follows;

q=pots⋅(d1Ad2G+d1Gd2A+d1Cd2T+d1Td2C)r=potv⋅(d1Ad2C+d1Cd2A+d1Ad2T+d1Td2A+d1Gd2C+d1Cd2G+d1Gd2T+d1Td2G)i=poid⋅(d1Ad2A+d1Cd2C+d1Gd2G+d1Td2T)
 MathType@MTEF@5@5@+=feaafiart1ev1aaatCvAUfKttLearuWrP9MDH5MBPbIqV92AaeXatLxBI9gBaebbnrfifHhDYfgasaacH8akY=wiFfYdH8Gipec8Eeeu0xXdbba9frFj0=OqFfea0dXdd9vqai=hGuQ8kuc9pgc9s8qqaq=dirpe0xb9q8qiLsFr0=vr0=vr0dc8meaabaqaciaacaGaaeqabaqabeGadaaakeaafaqaaeWadaaabaGaemyCaehabaGaeyypa0dabaGaemiCaa3aaSbaaSqaaiabd+gaVjabdsha0jabdohaZbqabaGccqGHflY1daqadaqaaiabdsgaKnaaDaaaleaacqaIXaqmaeaacqWGbbqqaaGccqWGKbazdaqhaaWcbaGaeGOmaidabaGaem4raCeaaOGaey4kaSIaemizaq2aa0baaSqaaiabigdaXaqaaiabdEeahbaakiabdsgaKnaaDaaaleaacqaIYaGmaeaacqWGbbqqaaGccqGHRaWkcqWGKbazdaqhaaWcbaGaeGymaedabaGaem4qameaaOGaemizaq2aa0baaSqaaiabikdaYaqaaiabdsfaubaakiabgUcaRiabdsgaKnaaDaaaleaacqaIXaqmaeaacqWGubavaaGccqWGKbazdaqhaaWcbaGaeGOmaidabaGaem4qameaaaGccaGLOaGaayzkaaaabaGaemOCaihabaGaeyypa0dabaGaemiCaa3aaSbaaSqaaiabd+gaVjabdsha0jabdAha2bqabaGccqGHflY1daqadaqaaiabdsgaKnaaDaaaleaacqaIXaqmaeaacqWGbbqqaaGccqWGKbazdaqhaaWcbaGaeGOmaidabaGaem4qameaaOGaey4kaSIaemizaq2aa0baaSqaaiabigdaXaqaaiabdoeadbaakiabdsgaKnaaDaaaleaacqaIYaGmaeaacqWGbbqqaaGccqGHRaWkcqWGKbazdaqhaaWcbaGaeGymaedabaGaemyqaeeaaOGaemizaq2aa0baaSqaaiabikdaYaqaaiabdsfaubaakiabgUcaRiabdsgaKnaaDaaaleaacqaIXaqmaeaacqWGubavaaGccqWGKbazdaqhaaWcbaGaeGOmaidabaGaemyqaeeaaOGaey4kaSIaemizaq2aa0baaSqaaiabigdaXaqaaiabdEeahbaakiabdsgaKnaaDaaaleaacqaIYaGmaeaacqWGdbWqaaGccqGHRaWkcqWGKbazdaqhaaWcbaGaeGymaedabaGaem4qameaaOGaemizaq2aa0baaSqaaiabikdaYaqaaiabdEeahbaakiabgUcaRiabdsgaKnaaDaaaleaacqaIXaqmaeaacqWGhbWraaGccqWGKbazdaqhaaWcbaGaeGOmaidabaGaemivaqfaaOGaey4kaSIaemizaq2aa0baaSqaaiabigdaXaqaaiabdsfaubaakiabdsgaKnaaDaaaleaacqaIYaGmaeaacqWGhbWraaaakiaawIcacaGLPaaaaeaacqWGPbqAaeaacqGH9aqpaeaacqWGWbaCdaWgaaWcbaGaem4Ba8MaemyAaKMaemizaqgabeaakiabgwSixpaabmaabaGaemizaq2aa0baaSqaaiabigdaXaqaaiabdgeabbaakiabdsgaKnaaDaaaleaacqaIYaGmaeaacqWGbbqqaaGccqGHRaWkcqWGKbazdaqhaaWcbaGaeGymaedabaGaem4qameaaOGaemizaq2aa0baaSqaaiabikdaYaqaaiabdoeadbaakiabgUcaRiabdsgaKnaaDaaaleaacqaIXaqmaeaacqWGhbWraaGccqWGKbazdaqhaaWcbaGaeGOmaidabaGaem4raCeaaOGaey4kaSIaemizaq2aa0baaSqaaiabigdaXaqaaiabdsfaubaakiabdsgaKnaaDaaaleaacqaIYaGmaeaacqWGubavaaaakiaawIcacaGLPaaaaaaaaa@CEAE@

The contribution to the number of observed transitions is now given by qi+q+r
 MathType@MTEF@5@5@+=feaafiart1ev1aaatCvAUfKttLearuWrP9MDH5MBPbIqV92AaeXatLxBI9gBaebbnrfifHhDYfgasaacH8akY=wiFfYdH8Gipec8Eeeu0xXdbba9frFj0=OqFfea0dXdd9vqai=hGuQ8kuc9pgc9s8qqaq=dirpe0xb9q8qiLsFr0=vr0=vr0dc8meaabaqaciaacaGaaeqabaqabeGadaaakeaadaWcaaqaaiabdghaXbqaaiabdMgaPjabgUcaRiabdghaXjabgUcaRiabdkhaYbaaaaa@341E@ and the contribution to the number of observed transversions by ri+q+r
 MathType@MTEF@5@5@+=feaafiart1ev1aaatCvAUfKttLearuWrP9MDH5MBPbIqV92AaeXatLxBI9gBaebbnrfifHhDYfgasaacH8akY=wiFfYdH8Gipec8Eeeu0xXdbba9frFj0=OqFfea0dXdd9vqai=hGuQ8kuc9pgc9s8qqaq=dirpe0xb9q8qiLsFr0=vr0=vr0dc8meaabaqaciaacaGaaeqabaqabeGadaaakeaadaWcaaqaaiabdkhaYbqaaiabdMgaPjabgUcaRiabdghaXjabgUcaRiabdkhaYbaaaaa@3420@ That is, the contribution is given by normalized posterior probabilities. The ML estimate of the distance can now be computed by maximizing the likelihood function using the new observed number of mutational events.

**Example of calculations **Assume that there are two sequences of length 101. One of the sequences has an R in one of the sites and the other sequence has an A in the same site. In the other 100 positions there are 4 transversions and 10 transitions. For simplicity we compute the model parameters without using a fixed transition/transversion ratio, i.e. *p*_*otv *_= 0.04, *p*_*ots *_= 0.1, and *p*_*oid *_= 0.86. Continuing we get that *q *= *p*_*ots*_·0.25, *r *= *p*_*otv*_·0, and *i *= *p*_*oid*_·0.25. Thus the total number of observed transitions becomes 10.106.

#### Resolving ambiguity Symbols by nearest neighbor

In the general method above the resolutions of ambiguities were given a uniform prior. Now we will adjust the distribution to reflect that ambiguous sites evolve according to the same evolutionary process as the rest of the sequence. In practice, we adjust the distribution such that the probability of observing the mutational events between the resolutions of the ambiguity and the nucleotide in the closest other sequence coincide with the overall probability of observing the same mutational events. Therefore, we refer to the method as resolving by nearest neighbor. We introduce the method by a simple example under Kimura's two-parameter model.

Let *s*_1 _be a sequence with ambiguity Symbols that we would like to resolve and let *s*_2 _be the closest other sequence. We first compute the probabilities of observing the different events using the unambiguous sites, i.e., *p*_*otv*_, *p*_*ots*_, and *p*_*oid*_. Thereafter, the distribution of each ambiguity symbol in *s*_1 _is changed to reflect the probabilities of mutating from the nucleotide in *s*_2 _to each of the resolutions in *s*_1_. For simplicity we do this only if the nucleotide in *s*_2 _is unambiguous and one of the possible resolutions of the ambiguity in *s*_1_. For example, let the ambiguity symbol in *s*_1 _be R, i.e. {*p*_*A *_= 0.5, *p*_*C *_= 0, *p*_*G *_= 0.5, *p*_*T *_= 0}, and let the unambiguous symbol in *s*_2 _be A (which is one of the resolutions of R). Then the distribution (For models such as the Tamura-Nei model for which the basic distribution is not uniform the original distribution of the ambiguity should be included when Computing the new distribution of the ambiguity symbol.) of the ambiguity symbol is updated as

{pA=poidpoid+pots,pC=0,pG=potspoid+pots,pT=0}.
 MathType@MTEF@5@5@+=feaafiart1ev1aaatCvAUfKttLearuWrP9MDH5MBPbIqV92AaeXatLxBI9gBaebbnrfifHhDYfgasaacH8akY=wiFfYdH8Gipec8Eeeu0xXdbba9frFj0=OqFfea0dXdd9vqai=hGuQ8kuc9pgc9s8qqaq=dirpe0xb9q8qiLsFr0=vr0=vr0dc8meaabaqaciaacaGaaeqabaqabeGadaaakeaacqGG7bWEcqWGWbaCdaWgaaWcbaGaemyqaeeabeaakiabg2da9maalaaabaGaemiCaa3aaSbaaSqaaiabd+gaVjabdMgaPjabdsgaKbqabaaakeaacqWGWbaCdaWgaaWcbaGaem4Ba8MaemyAaKMaemizaqgabeaakiabgUcaRiabdchaWnaaBaaaleaacqWGVbWBcqWG0baDcqWGZbWCaeqaaaaakiabcYcaSiabdchaWnaaBaaaleaacqWGdbWqaeqaaOGaeyypa0JaeGimaaJaeiilaWIaemiCaa3aaSbaaSqaaiabdEeahbqabaGccqGH9aqpdaWcaaqaaiabdchaWnaaBaaaleaacqWGVbWBcqWG0baDcqWGZbWCaeqaaaGcbaGaemiCaa3aaSbaaSqaaiabd+gaVjabdMgaPjabdsgaKbqabaGccqGHRaWkcqWGWbaCdaWgaaWcbaGaem4Ba8MaemiDaqNaem4CamhabeaaaaGccqGGSaalcqWGWbaCdaWgaaWcbaGaemivaqfabeaakiabg2da9iabicdaWiabc2ha9jabc6caUaaa@68AE@

**Figure 3:**
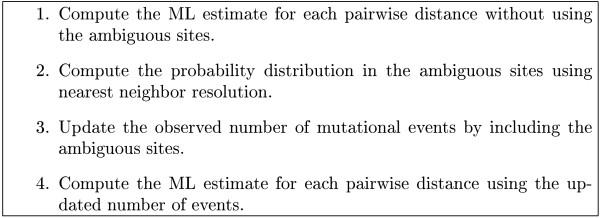
**Estimation when ambiguities are present**. Overview of the combined method, the default in fastdist, for estimating pairwise distances when ambiguity symbols are present.

After having updated the distribution of all ambiguous sites our general technique above can be applied to compute the ML estimate of the distance. Thus, using the general method and the nearest neighbor resolution technique together, we end up with the method in Figure 3.

#### Generating the simulated data and evaluating the ambiguity methods

To evaluate the ambiguity methods we generated data as follows. Model trees were generated through a random birth-death process using code from the software package [12]. These trees where then made non-ultra metric, i.e., root to leaf paths where made to vary in length, by multiplying the edge lengths with a random number in the interval [1/16,1]. Subsequently, sequence data was generated according to Kimura's two-parameter model using the Seq-Gen program [13]. Altogether we generated test data with all combinations of trees of sizes 10, 20, 30, 40, 50, 60, 70, 80, 90, 100 and sequences of lengths 500, 1000, 2000, 4000. To get statistically sound results, we generated 20 data sets for each such test size. Finally, for each data set, we changed nucleotides into ambiguity Symbols, uniformly at random but so that the resolutions of the ambiguity symbol contained the original nucleotide and only one more resolution, e.g., an A was allowed to change into an R. We performed tests with 1%, 2%, and 5% ambiguities inserted at random.

Four different measures were used to compare the accuracy of the methods. The first three, Euclidean distance, the max norm, and the absolute sum of all differences, measured matrix norms between the data with the ambiguities and the data without the ambiguities. For the fourth measure, we computed a tree using Neighbor Joining and compared the normalized Robinson-Foulds (RF) distance between the model tree and the tree given by N.J. This fourth measure is however not a good test, since it is based on the accuracy of the NJ algorithm which is a heuristic; for some cases, NJ did even better on data with ambiguities than on the original data.

The average over all 20 datasets was computed across each combination of measure and test size. In Figure 1, the test size with sequence length 1000 and 2% ambiguities is shown. All additional tests can be viewed in the supplementary material. However, the methods relative behavior is the same for all test Parameters. It can be seen that accuracy of our combined approach is superior to that of the three other methods.

### A novel algorithm for Computing the number of mutational events

Our novel algorithm takes two DNA sequences as input and counts the number of transitions and transversions (it can be extended to count the number of purine-transitions and pyrimidine-transitions). It is based on an elaborate divide and conquer and bit-fiddling strategy, i.e., the problem is divided into subproblems which are solved and Solutions are combined using the bitwise representation and low-level tricks. In the present case, a compact binary coding is adopted which allows for 64 nucleotides to be packed into 128 bits. Moreover, since most modern day processors have 128 bit registers (The 128 bit registers in Intel's processors are known as SSE2 and in Apple's processors Altivec.) it is possible to operate on all 64 nucleotides in parallel. Exploiting the 128 bit registers and elaborate bit-fiddling the algorithm computes the number of mutational events of more than 64 nucleotides at a time.

The algorithm is presented in three steps. First the binary coding is described, then it is shown how to compute the mutational events of 64 nucleotides at a time, using bit-fiddling. Finally, the algorithm is extended to achieve an even higher speed-up.

#### Binary coding

To fit 64 nucleotides into 128 bits each nucleotide is represented by two bits as described in Table 3. This coding has been chosen to simplify the comparison of nucleotides. Consider two sequences *u *and *v *both with 64 nucleotides and let *r *= *u *⊕ *v *be their bitwise xor. We notice that for each site in which *u *and *v *differ the associated block of two bits in *r *can be used to decide whether a transition or transversion has occurred, see Table 4 and Table 5. In particular, notice that the left bit in each block is 1 only if a transversion has occurred. Therefore, after evaluating

**Table 3:** Binary representation of nucleotides.

**Nucl**.	**Coding**
A	00
C	11
G	01
T	10

**Table 4:** The bitwise xor of nucleotides.

⊕	**A**	**C**	**G**	**T**
**A**	00	11	01	10
**C**		00	10	01
**G**			00	11
**T**				00

**Table 5:** The binary representation mutational events.

**Change**	**xor**
equal	00
transition	01
transversion	10,11

tv←(r>>1)︸rightshift one bit&m1︸mask…0101
 MathType@MTEF@5@5@+=feaafiart1ev1aaatCvAUfKttLearuWrP9MDH5MBPbIqV92AaeXatLxBI9gBaebbnrfifHhDYfgasaacH8akY=wiFfYdH8Gipec8Eeeu0xXdbba9frFj0=OqFfea0dXdd9vqai=hGuQ8kuc9pgc9s8qqaq=dirpe0xb9q8qiLsFr0=vr0=vr0dc8meaabaqaciaacaGaaeqabaqabeGadaaakeaacqWG0baDcqWG2bGDcqGHqgcRdaagaaqaaiabcIcaOiabdkhaYjabg6da+iabg6da+iabigdaXiabcMcaPaWcbaGaeeOCaiNaeeyAaKMaee4zaCMaeeiAaGMaeeiDaqNaee4CamNaeeiAaGMaeeyAaKMaeeOzayMaeeiDaqNaeeiiaaIaee4Ba8MaeeOBa4MaeeyzauMaeeiiaaIaeeOyaiMaeeyAaKMaeeiDaqhakiaawIJ=aiabcAcaMmaayaaabaGaemyBa02aaWbaaSqabeaacqaIXaqmaaaabaGaeeyBa0MaeeyyaeMaee4CamNaee4AaSMaeSOjGSKaeGimaaJaeGymaeJaeGimaaJaeGymaedakiaawIJ=aaaa@60AD@

*tv *is a string in which each block is 01 if a transversion has occurred and 00 otherwise (the bitwise operations are listed in Table 6). Similarly after evaluating

**Table 6:** List of bitwise operations.

**Symbol**	**Meaning**
*x *>> *I*	Rihtshift *x I *bits.
*x *&*y*	Bitwise and of *x *and *y*
*x *⊕ *y*	Bitwise xor of *x *and *y*
~ *x*	Bitwise negation of *x*
*m*^1^	Mask where every other bit is 1.
*m*^*I*^	Mask where every other block of *I *= 2^*i *^bits are ones.

ts←(r&m1)&(~tv)︸bitwise negation of tv
 MathType@MTEF@5@5@+=feaafiart1ev1aaatCvAUfKttLearuWrP9MDH5MBPbIqV92AaeXatLxBI9gBaebbnrfifHhDYfgasaacH8akY=wiFfYdH8Gipec8Eeeu0xXdbba9frFj0=OqFfea0dXdd9vqai=hGuQ8kuc9pgc9s8qqaq=dirpe0xb9q8qiLsFr0=vr0=vr0dc8meaabaqaciaacaGaaeqabaqabeGadaaakeaacqWG0baDcqWGZbWCcqGHqgcRcqGGOaakcqWGYbGCcqGGMaGjcqWGTbqBdaahaaWcbeqaaiabigdaXaaakiabcMcaPiabcAcaMmaayaaabaGaeiikaGIaeiOFa4NaemiDaqNaemODayNaeiykaKcaleaacqqGIbGycqqGPbqAcqqG0baDcqqG3bWDcqqGPbqAcqqGZbWCcqqGLbqzcqqGGaaicqqGUbGBcqqGLbqzcqqGNbWzcqqGHbqycqqG0baDcqqGPbqAcqqGVbWBcqqGUbGBcqqGGaaicqqGVbWBcqqGMbGzcqqGGaaicqWG0baDcqWG2bGDaOGaayjo+daaaa@5D56@

*ts *is a string in which each block is 01 if a transition has occurred and 00 otherwise.

#### Counting the number of events in a 128-bit word

After having separated the transition and transversion events as above, we are left with the task of Computing the number of ones in *ts *and *tv*, respectively. At first appearance this might seem like a simple task. Unfortunately though, there is no immediate way of counting the number of ones in a Computer word. Instead this is done using simple operations such as addition and logical bit-wise operations.

Consider the 64 blocks of *ts *and think of them as a sequence of integers such that each block is the binary representation of the integer. For instance, suppose that the blocks are (..., 01, 00, 01, 01) then the corresponding integer sequence is (..., 1, 0, 1, 1) and the number of transitions is the sum of these integers. The regular way of Computing this sum is to sequentially iterate over all integers and add them using 63 additions. However, with divide and conquer and bit-fiddling the sum can be computed using no more than 6 additions!

**Figure 4:**
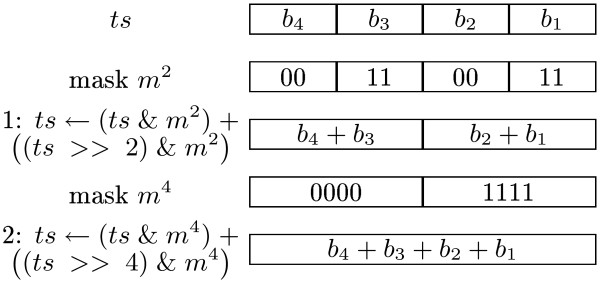
**Addition through divide and conquer**. Computing the sum of four 2-bit integers in parallel using bit-fiddling.

In Figure 4, the divide and conquer idea is demonstrated on the first four blocks of *ts*, (..., *b*_4_, *b*_3_, *b*_2_, *b*_1_). Notice that the sum is computed as (*b*_4 _+ *b*_3_) + (*b*_2 _+ *b*_1_), where the two parentheses are computed in parallel. Moreover, after evaluating the first line the block size has been increased from two to four bits and in the second line to eight bits. Hence, returning to the example, the sum is computed as

(1,0,1,1) → (1,2) → 3.

**Figure 5:**
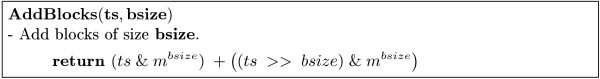
**Subroutine AddBlocks**. This subroutine takes a 128 bit word **ts **divided into blocks of size **bsize **as input and adds every two blocks together into a new block of size 2·**bsize**.

The divide and conquer algorithm then proceeds by adding blocks of size 8, 16, 32, and finally 64. Thus the number of transitions in 64 nucleotides is counted by calling the subroutine **AddBlocks**, in Figure 5, six times.

#### Optimizing the counting for many words

Above we described how to process 64 nucleotides at a time. Here the algorithm is extended to achieve even higher speed-up for sequences longer than 64 nucleotides. As above, we introduce the idea using a simple example.

Consider two sequences of length 192 and let *ts*_*i *_be the transition sequences of the 64 positions (64 × (*i *- 1), 64 × *i*). For example, we may have *ts*_1 _= (..., 1, 0, 1, 1), *ts*_2 _= (..., 0, 0, 1, 0), and *ts*_3 _= (..., 1, 0, 1, 0). Adding these three sequences as *ts *← *ts*_1 _+ *ts*_2 _+ *ts*_3 _then we get *ts *= (..., 2, 0, 3, 1), i.e., each block in *ts *contains the number of differences in three positions. Thereafter, the total number of differences can be computed by calling **AddBlocks **6 times on *ts*. Notice how this approach is a factor 3 faster then the sequential approach which requires 18 calls to **AddBlocks**.

Above we utilized that the blocks of *ts *are of size two and hold integers ≤ 3. But why stop here? After each call to **AddBlocks **the block size is doubled and it is possible to fit even bigger integers in each block. For example, after calling *ts *← **AddBlocks**(*ts*, 2) each block can hold integers ≤ 15. Therefore, when the DNA sequences contain 384 nucleotides, the following can be done

*ts *← **AddBlocks**(*ts*_1 _+ *ts*_2 _+ *ts*_3_, 2) +

**Figure 6:**
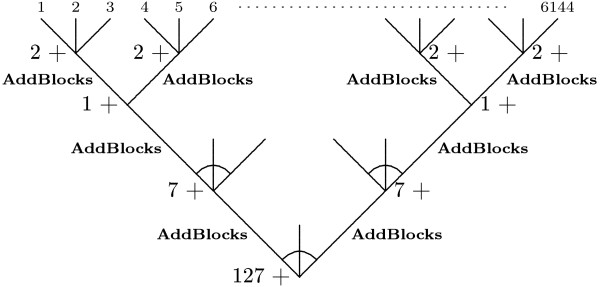
**Counting the number of mutational events**. Computing the number of events in 64 × 6144 positions. Each inner node represents the addition of a number of 128 bit words divided into blocks, e.g., 7+ denotes that 7 additions are used to add 8 128-bit words. Along each edge a call to **AddBlocks **is performed to double the block size. Thus in the nodes with 2+ the block size is 2, in the nodes with 1+ the block size is 4, in the nodes with 7+ the block size is 8, and in 127+ the block size is 16. Finally, another three calls to **AddBlocks **have to be performed to get all events into one single block.

**AddBlocks**(*ts*_4 _+ *ts*_5 _+ *ts*_6_, 2),

i.e., each block of *ts *is of size 4 and contains the number of transitions in 12 positions. In Figure 6, this idea has been extended such that the number of ones in 64 × 6144 positions are computed using 6143 additions and 2048 = 128 × 8 × 2 calls to **AddBlocks**. Notice how each subtree with six leaves describes the computation in which 6 sequences are added as above.

## Availability and requirements

The algorithm is implemented in C++ and is available for download without restrictions from . The current implementation only works on Intel processors with support for SSE2. Moreover, gcc 3.X is required to compile the source code. All supplementary material is available for download.

## Authors' contributions

IE did all implementations. JL and IE wrote the paper. Both authors have approved the final version of the manuscript.
